# Investigating the moral compensatory effect of unethical pro-organizational behavior on ethical voice

**DOI:** 10.3389/fpsyg.2023.1159101

**Published:** 2023-06-01

**Authors:** Fubin Xia, Ping Lu, Lifang Wang, Jiangdong Bao

**Affiliations:** ^1^School of Economics and Management, Hanshan Normal University, Chaozhou, China; ^2^School of Education Science, Hanshan Normal University, Chaozhou, China; ^3^School of Business Administration, Dongbei University of Finance and Economy, Dalian, China

**Keywords:** unethical pro-organizational behavior, moral ownership, ethical voice, benevolent leadership, moral compensation

## Abstract

**Introduction:**

Unethical pro-organizational behavior (UPB) can hinder the development of the organization. The existing literature on UPB rarely examines whether and how employees remedy such ethical misconduct after they have committed it. Based on moral compensation theory and social exchange theory, this study explores the self-moral compensation process of employees who engage in UPB.

**Methods:**

Specifically, we adopt a moderated mediating model to examine how and when UPB facilitates ethical voice. We tested our theoretical model using data from 415 full-time employees in Chinese companies, which we obtained via a three-stage questionnaire.

**Results:**

The results of the regression analysis revealed that UPB has a significant positive effect on ethical voice, and that moral ownership plays a mediating role between UPB and ethical voice. Furthermore, the results support the moderating role of benevolent leadership in the positive direct effect of UPB on ethical voice, and the positive indirect effect of UPB on ethical voice via moral ownership. When benevolent leadership is strong, the direct effect of UPB on ethical voice and indirect mediating effect of moral ownership are both significantly positive, whereas neither are significant when benevolent leadership is weak.

**Discussion:**

These findings show the ethical compensation effect of UBP on ethical voice and provide a novel and comprehensive understanding of the consequences of UPB. They also have significant value for ethical practices in managing employee (mis)behavior.

## 1. Introduction

In today’s increasingly technologically innovative world, employees sometimes take the initiative or are forced to resort to unethical behavior to aid the company’s survival. This kind of unethical behavior with altruistic motives for the benefit of the organization is called unethical pro-organizational behavior (UPB; Umphress et al., 2010; Umphress and Bingham, 2011). Although employees engage in UPB with the intention of promoting organizational effectiveness, the unethical nature of their behavior can cause potential and real harm to the organization and its members (Umphress and Bingham, 2011), ultimately hindering or undermining development of organization (e.g., Huang et al., 2022; Kelebek and Alniacik, 2022). As a result, after committing UPB, although employees usually attempt to ethically justify their actions (Zhang et al., 2022), they may also experience negative problems such as guilt (Tang et al., 2020; Wang et al., 2022), cognitive dissonance (Umphress and Bingham, 2011), emotional conflict and anxiety (Liu et al., 2021), and stress at work (Chen et al., 2022). These common reactions suggest that employees who have committed UPB realize the harmfulness of their actions—that is, they recognize that they have engaged in inappropriate actions (Graham et al., 2020).

Individuals often seek to correct their mistakes and make amends after they have done something wrong. Recent research has verified that employees who make mistakes engage in role model behavior (Bonner et al., 2017), organizational citizenship behavior (Tang et al., 2020), and voice behavior (Wang et al., 2022). Thus, it is reasonable to believe that employees who make ethical mistakes for the sake of the organization will also attempt to correct them. If so, how and when do they make amends for such ethical mistakes? Extant literature on UPB does not provide an answer to this question. Most existing studies focus on exploring the antecedents of UPB from the perspectives of social identity (e.g., Effelsberg et al., 2014; Johnson and Umphress, 2019; Baur et al., 2020), social exchange (e.g., Babalola et al., 2021; Nguyen et al., 2021; Farasat and Azam, 2022), social learning (e.g., Zhang et al., 2018; Fehr et al., 2019; Lian et al., 2022), and social cognition (e.g., Lee et al., 2019; Valle et al., 2019; Sheedy et al., 2021; see Mo et al., 2022), whereas the potential consequences of UPB remain poorly understood (Wang et al., 2022). Against this background, this study explores the processes inherent in self-remediation of employees who engage in UPB, and the conditions under which it occurs, in order to fill the gap in the existing UPB literature.

Moral compensation theory provides a theoretical framework for exploring the processes involved in the self-remediation of employees who engage in UPB. According to moral compensation theory, after committing an unethical act, people tend to engage in ethical or pro-social behavior to atone for their transgressions (Nisan and Horenczyk, 1990; Cornelissen et al., 2013). Based on moral compensation theory, we consider the process of self-remediation of employees who have engaged in UPB as a moral compensation process, and argue that after committing UPBs, they will subsequently engage in moral compensation behaviors such as ethical behavior.

Ethical voice may be a means of providing ethical compensation for employees who engage in unethical acts to aid their organization. Ethical voice is a type of behavior performed by organizational members to enhance the ethical practices of their organization (Huang and Paterson, 2017; Lee et al., 2017). Although it may not directly compensate the victim, it can benefit society by enhancing the organization’s ethical practices (Lee et al., 2017; Zheng et al., 2021), thereby producing a greater alternative moral compensation effect. By identifying and addressing existing unethical issues within the organization (Lee et al., 2017; Zheng et al., 2021), ethical voice can contribute to a more ethical and inclusive organizational culture. As such, employees who engage in ethical voice after committing unethical pro-organizational behavior not only perform moral compensation but also discourage other organizational members from engaging in unethical behavior, especially when it serves the organization’s interests. Therefore, from the moral compensation perspective, we propose that after engaging in UPB, employees can develop a sense of moral responsibility or moral motivation to perform ethical behavior, namely moral ownership (Hannah et al., 2011), and, consequently, under its influence, they may make ethical voice to remedy their faults. This means that employees who engage in unethical behavior may make ethical voice through moral ownership to compensate for the moral faults arising from their UPB.

Social exchange theory (Blau, 1964) provides a theoretical basis for uncovering the conditions under which self-remediation occurs for employees who engage in unethical behavior to aid the organization. It has been shown that the social exchange relationship between leaders and subordinates has a significant impact on subordinates’ work attitudes and behaviors (e.g., Graen and Uhl-Bien, 1995; Martin et al., 2016). Therefore, from the perspective of social exchange between leaders and subordinates, we explore how leaders should treat ethically erring subordinates so as to promote their moral compensation.

Benevolent leadership based on Confucian culture, which emphasizes one’s own moral cultivation and leading subordinates with virtue (Farh and Cheng, 2000), may be conducive to promoting moral compensation for moral wrongdoers who commit UPB. Benevolent leadership is ethically sensitive and concerned with the ethical practices of the organization (Karakas and Sarigollu, 2012). Moreover, benevolent leaders can show individual, comprehensive, and long-term care to subordinates (Wang and Cheng, 2010), and provide subordinates with a safe psychological environment, opportunities to correct mistakes, and more task resources and support (Farh et al., 2008). Subordinates, in turn, show more gratitude and reward (e.g., through loyalty and obedience) to their leader in the reciprocal social exchange process (Chen et al., 2014; Li et al., 2018). Therefore, we contend that benevolent leadership can not only detect subordinates’ moral wrongdoing sensitively, but also help and support employees who have committed UPB toward self-ethical remediation. In other words, based on social exchange theory, benevolent leadership may be one of the boundary conditions for such employees to engage in moral remediation through ethical voice.

**Figure 1:**
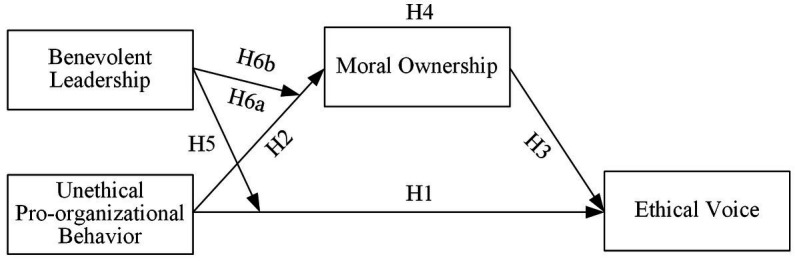
Hypothesized research model.

Overall, in order to explore the internal process of self-moral remediation of employees who commit UPB and the conditions under which it occurs, based on moral compensation and social exchange theory, our research adopts a moderated mediating model (see Figure 1) to investigate the moral compensatory effect of UPB on ethical voice, and has three research objectives: (1) to examine the impact of UPB on ethical voice; (2) to identify the mediating role of moral ownership in the effects of UPB on ethical voice; and (3) to test the moderating role of benevolent leadership in the direct effect of UPB on ethical voice, and in the indirect effect of UPB on ethical voice via moral ownership.

Examining the moral compensatory effect of UPB on ethical voice contributes to the literature on UPB in several ways. First, it extends existing knowledge of UPB by providing a new understanding of the consequences of UPB, which enables a more reasonable approach to managing employees who engage in unethical behavior for the sake of the organization. Second, it analyzes the underlying process by which UPB affects ethical voice from the perspective of moral ownership, and provides an in-depth understanding of this self-remediation process of employees who engage in UPB. Third, it examines the moderating role of benevolent leadership in strengthening the process of moral compensation, which not only bridges the gap between leadership literature and UPB literature, but also explores novel directions for research on leadership practices relating to UPB as a negative organizational behavior.

## 2. Theory and hypothesis development

### 2.1. UPB and ethical voice

Although people value morality, they often commit immoral acts because they cannot resist temptation (Jordan et al., 2011; Tang and Sutarso, 2013). According to moral compensation theory, people engage in positive behaviors such as moral or pro-social behaviors after committing unethical behavior, in order to compensate for their moral transgressions (Nisan and Horenczyk, 1990; Cornelissen et al., 2013). There are two motives underlying moral reparation behaviors. One is relational motivation, which restores one’s moral image in the eyes of others so as to avoid damage to one’s relationships and reputation (Bateson et al., 2006; Gino et al., 2011; Greene and Low, 2014). The second is the ego motive, which is to protect one’s sense of self-worth and restore the damaged moral self (Sachdeva et al., 2009; Gino, 2015; Bonner et al., 2017).

UPB is the intentional behavior of an employee in which core social values, moral customs, laws, and norms of proper behavior are violated to promote the effective operation of the organization or the effective work of its members (Umphress et al., 2010; Umphress and Bingham, 2011). As mentioned earlier, although UPB is pro-organizational in nature, it can harm others and society, and thus employees may realize they have performed unethical behavior even if they do it for the sake of the organization (Graham et al., 2020; Wang et al., 2022). Therefore, according to moral compensation theory, an employee who commits UPB may next commit moral compensation behavior as an act of moral reparation.

Ethical voice—an ethical behavior that is critical to the survival and growth of an organization—involves members publicly speaking out and opposing existing ethically inappropriate behaviors, procedures, and policies within the organization for the purpose of improving the organization’s ethical status (Huang and Paterson, 2017; Lee et al., 2017). Ethical voice helps the organization identify and address existing unethical issues and practices in a timely manner to avoid serious problems (Lee et al., 2017; Zheng et al., 2021). From this, it can be presumed that ethical voice may be one of the ethical compensation options for erring employees.

Ethical voice can help errant employees who engage in UPB to achieve relational motivation. When employees speak out against or oppose unethical practices and issues in the organization, they often invite resentment and retaliation, and even interpersonal conflict (Lee et al., 2017). These risks mean that employees have to pay a higher price for making ethical voice (Paterson and Huang, 2019; Zheng et al., 2022). Employees’ high-cost ethical voice behaviors that involve sacrificing themselves for the good of the organization and its members can gain the approval and respect of others (supervisors and colleagues), thus restoring their damaged ethical image and avoiding punishment from others. Furthermore, research has shown that ethical behavior can enhance sense of self-worth and help individuals restore their damaged moral self (Sachdeva et al., 2009; Jordan et al., 2011). Thus, the high-value and high-risk ethical behavior of ethical voice by employees who engage in UPB can restore their moral self that has been destroyed by their wrongdoings, and thus help them achieve self-motivation. The above analysis suggests that errant employees who have committed UPB may engage in ethical voice behaviors to make amends. Therefore, the following hypothesis is proposed:

*H1*: UPB has a positive effect on ethical voice.

### 2.2. The mediating role of moral ownership

An individual may develop moral ownership when he or she believes that he or she should take moral responsibility for his or her actions. Moral ownership is the sense of motivation held by an individual to support morality or to act ethically for the environment he or she is in (Hannah and Avolio 2010), and reflects the degree of moral responsibility an individual feels he or she should take for his or her own behavior, those of others around them, their organization, or another collective (Hannah et al., 2011).

UPB is a behavior that destroys an employee’s moral self (Wang et al., 2022). However, individuals seek consistency or integrity of the self, and when an individual’s behavior is inconsistent with the self, the individual will be motivated to adjust his or her behavior to perform behavior consistent with the self, thus regaining a sense of self-completeness (Gollwitzer et al., 1982; Pyszczynski et al., 2004). Therefore, when an employee performs UPB behavior and thus experiences his or her moral self as incomplete, he or she will have the moral motivation to change their behavior, that is, form a sense of moral responsibility to restore the damaged moral self. Furthermore, when an employee realizes that his or her UPB violates ethical standards or that the behavior causes harm to others or society, he or she usually believes that he or she should be responsible for his or her wrongdoing and feels moral responsibility for his or her next ethical action (Jones and Ryan, 1997)—in other words, he or she experiences moral ownership. Based on the above analysis, it can be predicted that UPB may increase the moral ownership of erring employees, which leads to the following hypothesis:

*H2*: UPB has a positive effect on moral ownership.

Erring employees may choose ethical voice as ethical compensatory behaviors after moral ownership is promoted. Existing research provides evidence for this possibility, and moral ownership has been found to be one of the driving forces that motivate individuals to behave ethically (e.g., Jino and Dyaram, 2019; Ogunfowora et al., 2021). Based on these research findings, it is then reasonable to believe that ethical voice, as an ethical behavior that enhances the current state of organizational morality, is likely to be chosen as a means of moral compensation by employees who engage in UPB motivated by moral ownership. More importantly, moral ownership (moral responsibility) leads individuals to be willing to take risks or make personal sacrifices to protect the moral self they own and value (Ogunfowora et al., 2021). As mentioned earlier, ethical voice is a self-sacrificial risk-taking moral behavior. From this, it can be hypothesized that, in cases where an individual’s moral self is damaged by his or her past UPB, moral ownership is likely to make erring employees willing to protect as well as restore their moral self through high-cost and high-risk ethical voice. The above two aspects of the analysis suggest that moral ownership may have a positive impact on ethical voice, and, in conjunction with hypothesis H2, we propose the following hypotheses:

*H3*: Moral ownership has a positive effect on ethical voice.

*H4*: Moral ownership mediates the relationship between UPB and ethical voice.

### 2.3. The moderating role of benevolent leadership

Benevolent leadership is a form of leadership based on Chinese Confucianism, whose core idea is that, based on benevolent reciprocity norms, the leader exchanges paternalistic benevolence for the childlike respect, loyalty, and obedience of his subordinates, thus achieving effective leadership over them (Farh and Cheng, 2000; Cheng et al., 2004). In leadership practice, this is expressed in the individual, comprehensive, and permanent care of the leader for the work and life of his subordinates—as a parent cares for his or her children (Farh and Cheng, 2000; Chen et al., 2014)—and this promotes positive work attitude and behavior of subordinates for the sake of the organization (e.g., Chan and Mak, 2012; Tang and Naumann, 2015; Gumusluoglu et al., 2017; Lin et al., 2018).

Following the way benevolent leaders treat their subordinates, we predict that the use of benevolent leadership by supervisors for employees who engage in UPB may facilitate their self-redress. First, when a benevolent leader demonstrates personalized and targeted care for the work and life of his or her subordinates, the subordinates are often grateful and willing to repay their supervisors through dedication and sacrificing ego (Farh et al., 2008). Therefore, when an employee who engages in UPB receives benevolent leadership from his or her supervisor, he or she may make self-sacrificing ethical voice for the purpose of repaying the favor. Second, benevolent leaders have a “compassionate and forgiving” side (Chua et al., 2008)—understanding, forgiving, and protecting subordinates, and not openly blaming and criticizing employees who commit wrongdoing (Farh et al., 2008). Therefore, benevolent supervisors will guide employees who engage in UPB and protect their image. Such forgiveness and tolerance would encourage errant subordinates to accept responsibility and take risks to correct their mistakes by making ethical voice. Third, a benevolent leader will provide the necessary resources, help, and support to subordinates when they encounter difficulties in their work (Cheng et al., 2004; Farh et al., 2008), creating the necessary external support conditions for the errant employee to make ethical voices to correct their mistakes. Finally, the benevolent leadership of supervisors creates a working atmosphere of mutual trust and psychological security for subordinates (Wang and Cheng, 2010), which, to a certain extent, dispels errant employees’ internal concerns about the possible risks of making ethical voice. The above analysis suggests that benevolent leadership may reinforce the influence of UPB on ethical voice. We propose the following hypothesis:

*H5*: Benevolent leadership positively moderates the relationship between UPB and ethical voice.

Benevolent leadership may also enhance the impact of UPB on moral ownership. When employees commit UPB, instead of criticizing and blaming them, their supervisors treat them with tolerance and provide them with guidance, help, and support (Chua et al., 2008; Farh et al., 2008). As a result, the guilt and remorse they originally felt as a result of their UPB may become stronger, which in turn may lead to a higher sense of moral responsibility to compensate for their faults. Specifically, this may happen for two reasons. One is that the benevolence of the supervisor toward them magnifies the moral character of the leader in the eyes of the subordinates, and creates a glorious and majestic parental moral image in their minds (Farh and Cheng, 2000). Another reason is that, based on the reciprocal nature of social exchange, the benevolence of the leader can cause subordinates to feel indebted to him or her (Wang and Cheng, 2010). Because of this sense of indebtedness and the moral influence of the supervisor, these erring employees are more deeply aware of their mistakes and more willing to take responsibility for them, and thus develop a stronger moral motivation to remedy their faults. In other words, the sense of reciprocal indebtedness to the supervisor and the supervisor’s moral contagion may further increase the moral ownership resulting from UPB. Accordingly, we propose the following hypotheses:

*H6a*: Benevolent leadership positively moderates the effect of UPB on moral ownership.

*H6b*: Benevolent leadership positively moderates the mediating role of moral ownership in the relationship between UPB and ethical voice.

Based on the above theoretical analysis, we propose the research model shown in Figure 1.

## 3. Materials and methods

### 3.1. Participants and procedures

In this study, we collected data using a questionnaire, which was conducted from May to August 2022. We focused on full-time employees in sales and service-related occupations, such as account specialists, customer service engineers, and salespeople, among others. This is because there are occupational differences in the occurrence of UPB, and employees who have direct contact with customers have more opportunities to engage in such behavior (Cheng et al., 2022). We used a convenience sampling technique to select eight specific companies in the Anhui and Heilongjiang provinces of China. These companies, which were in the real estate, information and financial services, and insurance industries, were chosen because of their close relationship with our research team, which ensured a smooth implementation of the survey.

We first contacted the HR managers of the eight companies. We explained the purpose and content of the study to gain their cooperation and support, and verified with them the occurrence of UPB in their companies. After their consent was obtained, we developed a data collection timeline with them. Before the questionnaire was distributed, we asked the HR managers to randomly select employees in sales and service-related positions from the company employee list and gather them in the conference room. We then gave detailed instructions and explanations about the study to the potential respondents and answered their questions. In order to allay their concerns and increase their interest in participating, they were then informed that the survey was voluntary and anonymous, and were assured that their information would be kept strictly confidential and used only for academic research. Finally, we recruited 550 employees who volunteered to participate in the survey.

We used a three-stage questionnaire with a two-week time interval for data collection to reduce common method bias arising from the use of self-reported questionnaires for measuring research variables in this study. Each employee who volunteered for the survey was assigned a three-digit numeric code (e.g., 016) to ensure that the three rounds of survey data could be matched. At time 1, with the help of the companies’ HR departments, a total of 550 questionnaires with three-digit numerical codes were distributed to corresponding matched employees who were asked to provide their demographic information and UPB. After eliminating invalid questionnaires, we obtained a total of 525 questionnaires in this round of survey. Two weeks later, we sent matching questionnaires to the remaining 525 employees based on their three-digit numerical codes to collect information about their direct supervisor’s benevolent leadership and their moral ownership, and received 480 valid questionnaires. At time point 3, we also administered matching questionnaires based on the three numerical codes of the remaining employees to measure their ethical voice, and obtained 415 valid questionnaires. Finally, after matching three rounds of measurement data by the three-digit numerical codes, we obtained a total of 415 valid and complete questionnaires for testing our theoretical model.

Among these 415 survey respondents, 51.6% were male; 65.3% were under 35 years old; 47.7% had a bachelor’s degree or higher; and 47.4% had less than 3 years of work experience.

### 3.2. Measures

The scales used in this study were derived from well-established scales that are widely used. To ensure the reliability and validity of the English scales in the Chinese context, we followed standard translation and back-translation procedures by Brislin (1986), and had them reviewed by professionals. All scale items were scored on a five-point Likert scale (1 = strongly disagree, 5 = strongly agree).

#### 3.2.1. UPB

In this study, UPB was assessed through a six-item scale developed by Umphress et al. (2010), with a Cronbach’s alpha of 0.935. A sample item from the UPB scale is “To help my organization, I exaggerated the truth about my company’s products or services to customers.”

#### 3.2.2. Benevolent leadership

A five-item scale developed by Fu et al. (2012), adapted from Cheng et al. (2000) scale, was used to test benevolent leadership, with one of the sample items being “My supervisor helps me in an emergency.” Cronbach’s alpha in this study was 0.906.

#### 3.2.3. Moral ownership

Hannah et al.’s (2011) three-item scale was used to measure moral ownership, with a Cronbach’s alpha of 0.793. One of the scale items is “I will not accept unethical behaviour from anyone in my organization.”

#### 3.2.4. Ethical voice

Ethical voice was measured via a four-item scale of Zheng et al. (2022), with a Cronbach’s alpha of 0.866. One of item of ethical voice scale is “I speak up in our company to stop others from behaving with a lack of integrity.”

#### 3.2.5. Control variables

Considering that demographic variables may have an impact on the study results, respondents’ age (1 = under 25 years, 2 = 26–35 years, 3 = 36–45 years, 4 = above 46 years), gender (1 = male, 2 = female), education (1 = high school and below, 2 = college, 3 = Bachelors, 4 = Masters and above), and working years (1 = under 1 years, 2 = 1–3 years, 3 = 4–6 years, 4 = 7–10 years, 5 = 10 years and above), were introduced as control variables in our analysis model.

## 4. Results

### 4.1. Descriptive statistics

The means, standard deviations, and correlation coefficients of the variables in this study are given in Table 1. There was a significant positive correlation between UPB and ethical voice (r = 0.266, *p* < 0.01), a significant positive correlation between UPB and moral ownership (r = 0.161, *p* < 0.01), and a significant positive correlation between moral ownership and ethical voice (r = 0.607, *p* < 0.01). These results lay the foundation for further testing the theoretical model of this study.

**Table 1:** Means, standard deviations, and correlation coefficients of the variables.

Variables	M	SD	1	2	3	4	5	6	7	8
1 Gender	1.480	0.500	1							
2 Age	2.200	0.948	−0.215^**^	1						
3 Education	2.330	0.948	0.036	−0.311^**^	1					
4 Years of work	2.940	1.610	−0.377^**^	0.635^**^	−0.339^**^	1				
5 UPB	2.718	1.187	−0.207^**^	−0.027	0.034	0.075	1			
6 BL	3.305	1.096	0.017	−0.041	0.043	−0.052	0.253^**^	1		
7 MO	3.684	0.897	−0.102^*^	0.114^*^	−0.014	0.106^*^	0.161^**^	0.405^**^	1	
8 EV	3.591	0.935	−0.094	0.110^*^	−0.101^*^	0.117^*^	0.266^**^	0.349^**^	0.607^**^	1

*N*, 415; ^*^p < 0.05, ^**^p < 0.01, ^***^p < 0.01; UPB, Unethical pro-organizational behavior; BL, Benevolent leadership; MO, Moral ownership; EV, Ethical voice.

### 4.2. Measurement model

First, the reliability of the variables UPB, moral ownership, moral voice, and benevolent leadership were tested by Cronbach’s alpha. The values were 0.935, 0.793, 0.866, and 0.906, respectively, and thus all exceed the recommended value of 0.7 (Nunnally, 1978). Therefore, the measurements of these variables have good reliability.

Then, Mplus 7.4 was used to conduct confirmatory factor analysis (CFA) on the four variables to analyze the discriminant validity of each variable. The results of the analysis in Table 2 show that the four-factor model outperforms the other models in all fit indicators (2 = 476.925, df = 129, RMSEA = 0.080, SRMR = 0.056, CFI = 0.933, TLI = 0.920), indicating that the four variables measured have good discriminant validity.

**Table 2:** Results of confirmatory factor analysis for the structural validity of variables.

Models	χ^2^	*df*	χ^2^*/df*	RMSEA	SRMR	CFI	TLI
Four-factor model (UPB, MO, EV, BL)	476.925	129	3.697	0.080	0.056	0.933	0.920
Three-factor model (UPB, MO + EV, BL)	626.185	132	4.744	0.095	0.065	0.904	0.889
Three-factor model (UPB + MO, EV, BL)	1029.988	132	7.803	0.128	0.148	0.826	0.798
Three-factor model (UPB, MO, EV + BL)	1292.128	132	9.789	0.146	0.128	0.775	0.739
Two-factor model (UPB + EV, MO + BL)	1665.067	134	12.426	0.166	0.170	0.703	0.661
Two-factor model (UPB + MO, EV + BL)	1733.012	134	12.933	0.170	0.179	0.690	0.646
One-factor model (UPB + MO + EV + BL)	2993.279	136	22.009	0.225	0.232	0.225	0.377

“+” Indicates factors combined; UPB, Unethical pro-organizational behavior; BL, Benevolent leadership; MO, Moral ownership; EV, Ethical voice.

Finally, we tested for common method bias by Harman single-factor test method. The results indicated that the percentage of variance explained by the first common factor was 36.353%, which was less than 40%, indicating that there was no serious common method bias. To further test for common method bias, in accordance with Podsakoff et al. (2003), we used the method of controlling the unmeasured potential method factors and establishing a two-factor model. The results of the analysis showed that the model fit indicators were not significantly better after adding common method bias as a latent variable to the CFA model to construct a common method factor model (ΔCFI = 0.032, ΔTLI = 0.031, ΔRMSEA = 0.018, ΔSRMR = 0.025), indicating that there was no significant common method bias.

### 4.3. Hypothesis tests

First, to test H1, ethical voice was set as the dependent variable; then, gender, age, education, and working years were added as control variables, and UPB was put into the regression equation as the independent variable. The linear regression results in Table 3 showed that UPB had a significant positive predictive effect on ethical voice (β = 0.210, *p* = 0.000), which supported H1.

**Table 3:** Regression results in this study.

Variable		Moral ownership		Ethical advice		
		Model 1	Model 2	Model 3	Model 4	Model 5
Control variable	Gender	−0.073	−0.095	−0.026	0.018	−0.043
	Age	0.097	0.089	0.079	0.021	0.072
	Education	0.021	0.023	−0.079	−0.091^*^	−0.077
	Years of work	0.012	0.029	0.008	0.001	0.022
Predictive variable	UPB	0.116^**^	0.004	0.210^***^	0.141^***^	0.118^**^
Mediator	Moral ownership				0.600^***^	
Moderator	BL		0.333^***^			0.271^***^
Interaction term	UPB * BL		0.090^**^			0.078^*^
*R*		0.206	0.457	0.302	0.639	0.440
*R-sq*		0.043	0.209	0.091	0.408	0.193
*F*		3.638^**^	25.331^***^	8.180^***^	46.922^***^	13.928^***^

*N* = 415; ^*^p < 0.05, ^**^p < 0.01, ^***^p < 0.01; UPB, Unethical pro-organizational behavior; BL, Benevolent leadership.

Second, on the basis of the main effect H1 holding, this study tested the mediating role of moral ownership via Baron and Kenny’s method (Baron and Kenny, 1986). The results in Table 3 show that, after putting the mediating variable moral ownership into the equation, UPB significantly and positively predicts moral ownership (β = 0.116, *p* < 0.01); therefore, hypothesis H2 is supported; As Hypothesis H3 predicted, moral ownership has a significant positive effect on ethical voice (β = 0.600, *p* < 0.001). Furthermore, the significant effect of UPB on ethical voice was attenuated (β = 0.141, *p* < 0.001). This suggested that moral ownership plays a partially mediating role in the relationship between UPB and ethical voice.

In addition, the mediating effect of moral ownership was further tested via the bootstrapping procedure (bootstrap = 5,000; Bolin, 2014). The results in Table 4 show that the indirect effect value of UPB influencing ethical voice through moral ownership is 0.070, contributing 33% to the total effect with a 95% confidence interval [LLCI = 0.017, ULCI = 0.121]; thus, hypothesis H4 is confirmed.

**Table 4:** Decomposition of total effect, direct effect, and indirect effect.

Paths and effects	Effect	BootSE	Boot 95% CI		Effectiveness ratio
Total effect (UPB → EV)	0.210	0.044	0.123	0.295	-
Direct effect (UPB → EV)	0.141	0.035	0.073	0.207	67%
Indirect effects (UPB → MO → EV)	0.070	0.026	0.017	0.121	33%

UPB, Unethical pro-organizational behavior; BL, Benevolent leadership; MO, Moral ownership; EV, Ethical voice.

**Figure 2:**
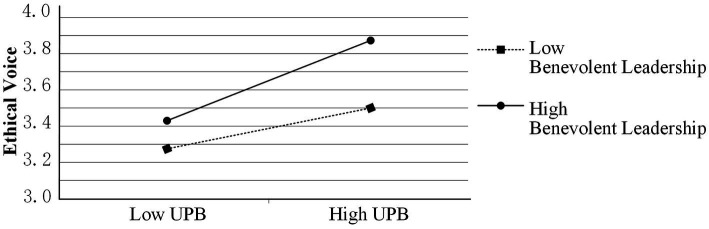
The moderating role of benevolent leadership in the relationship between UPB and ethical voice.

Third, the results of the analysis in Table 3 show that the interaction term between UPB and benevolent leadership has a significant positive effect on ethical voice (β = 0.078, *p* < 0.05), which indicates that benevolent leadership has a significant positive moderating effect on the relationship between UPB and ethical voice, confirming hypothesis H5. In order to analyze the moderating effect of benevolent leadership in more detail, further analysis was conducted on the relationship between UPB and ethical voice in the cases of strong benevolent leadership (+1SD) and weak benevolent leadership (−1SD) respectively. The results in Figure 2 show that when benevolent leadership is strong, UPB has a significant positive impact on ethical voice (β = 0.204, *p* = 0.000), whereas at weak benevolent leadership, UPB did not have a significant effect on ethical voice (β = 0.033, *p* > 0.05). This shows that strong benevolent leadership rather than weak benevolent leadership can promote ethical voice among employees who have engaged in UPB.

**Figure 3:**
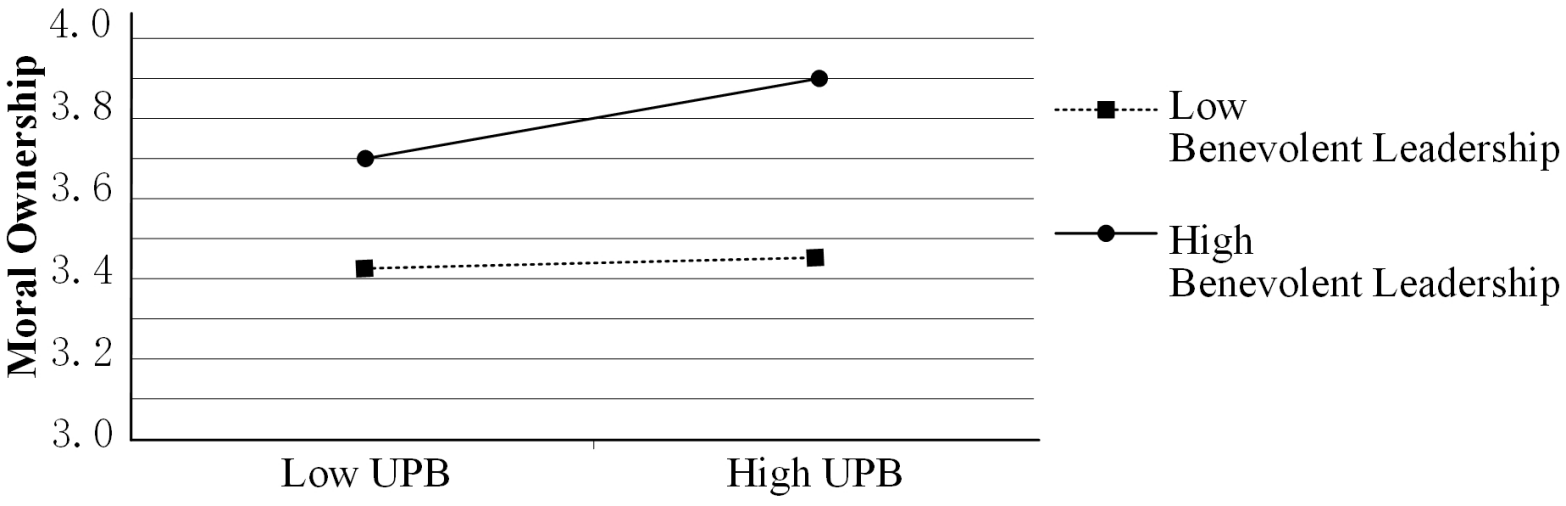
The moderating role of benevolent leadership in the relationship between UPB and moral ownership.

In addition, the results in Table 3 show that the interaction term between UPB and benevolent leadership has a significant positive effect on moral ownership (β = 0.090, *p* < 0.01), which confirms that benevolent leadership has a significant positive moderating effect in the relationship between UPB and moral ownership, thus validating hypothesis H6a. The relationship between UPB and moral ownership was analyzed separately for the two conditions of strong benevolent leadership (+1SD) and weak benevolent leadership (−1SD). The results in Figure 3 show that UPB has a significant positive effect on moral ownership when benevolent leadership is strong (β = 0.103, *p* < 0.05), but no significant effect on moral ownership when benevolent leadership is weak (β = −0.094, *p* > 0.05).

Finally, on the basis that hypothesis H6a holds, this study used bootstrapping to analyze the moderating effect of benevolent leadership on the mediating effect of moral ownership (Bolin, 2014). The results in Table 5 indicate that when benevolent leadership is strong, the mediating effect of moral ownership in the relationship between UPB and ethical voice is positive and significant (γ = 0.062, 95% confidence interval [0.017, 0.107]). When benevolent leadership is weak, the mediating effect of moral ownership is not significant (γ = −0.057, 95% confidence interval [−0.153, 0.032]). The index of moderated mediation was 0.054, 95% confidence interval [0.009, 0.103]. Hence, this result confirms hypothesis H6b.

**Table 5:** Mediated effect values of moral ownership at different levels of benevolent leadership.

Paths and effects	Effect	Standard error	Bootstrap’s 95% CI	
UPB (X) → MO (M) → EV (Y)				
Strong BL (+1SD)	0.062	0.023	0.017	0.107
Weak BL (−1SD)	−0.057	0.047	−0.153	0.032
Index of moderated mediation	0.054	0.024	0.009	0.103

UPB, Unethical pro-organizational behavior; BL, Benevolent leadership; MO, Moral ownership; EV, Ethical voice.

## 5. Discussion

This study examines the organizational phenomenon of self-remediation by employees engaging in UPB, based on moral compensation and social exchange theories (Blau, 1964; Nisan and Horenczyk, 1990). Survey data from 415 employees supported all of the study’s hypotheses. Previous research has shown a positive link between employee misconduct and good behavior (e.g., Bonner et al., 2017; Tang et al., 2020; Wang et al., 2022). Our study corroborates this finding and further reveals that the positive link is a moral compensatory one, driven by moral compensatory motivation. Specifically, we analyze how UPB influences ethical voice, based on moral compensation theory (Nisan and Horenczyk, 1990). The results show that UPB has a positive impact on ethical voice through moral ownership. This means that employees who have committed UPBs will develop moral ownership to remedy their faults, and, under its influence, will choose to make ethical voice for moral compensation. In addition, we find that benevolent leadership plays a positive moderating role in the moral compensation relationship between UPB and ethical voice. In other words, the existence of this relationship is related to supervisors’ leadership behaviors. Supervisors who adopt strong benevolent leadership not only help employees who have committed UPBs to rectify their faults directly through ethical voice, but also encourage them to choose ethical voice for indirect moral compensation through moral ownership. This finding aligns with previous research suggesting that when supervisors demonstrate benevolence toward their subordinates, the subordinates reciprocate with positive work attitudes and behaviors (Farh and Cheng, 2000; Chen et al., 2014; Li et al., 2018), indicating that the effect of benevolent leaders on subordinates’ behavior follows the principle of reciprocity in social exchange. Overall, these findings uncover the internal process and boundary conditions by which UPB influences ethical voice.

### 5.1. Theoretical contributions

First, this study uncovers a new consequence of UPB by revealing its positive relationship with ethical voice. Whereas most existing research on UPB consequences has focused on its negative effects (e.g., Baker et al., 2019; Lian et al., 2022), few studies have examined the positive behavioral changes triggered by UPB from a self-remediation perspective. Workplace behavior is complex and variable; employees may engage in harmful actions toward the organization after previous helpful acts (e.g., Klotz and Bolino, 2013; Yam et al., 2017; Loi et al., 2020) or engage in positive work behavior after harming the organization (e.g., Bonner et al., 2017; Tang et al., 2020; Wang et al., 2022). By exploring the moral compensation relationship between UPB and ethical voice from the perspective of sequential behavioral changes, this study provides new insights into the consequences of UPB and expands the scope of UPB research.

Second, this study not only finds moral compensatory effects of UPB, but also further explores its internal process and boundary conditions. The results indicate that moral ownership mediates the relationship between UPB and ethical voice, and that benevolent leadership moderates both the direct and indirect effects of UPB on ethical voice via moral ownership. Existing research explores the causes of moral ownership only in terms of organizational (Jino and Dyaram, 2019) and leadership factors (Ogunfowora et al., 2021; Su et al., 2022), with little attention to the role of individual factors, especially individual unethical behavior. Therefore, these new findings not only bridge gaps in moral ownership research but also further uncover the processes underlying the moral compensation effect of UPB by revealing the mediating role of moral compensation motives (moral ownership). Furthermore, leaders have a decisive influence on the behavior of their subordinates (e.g., Epitropaki et al., 2017), and our focus on benevolent leadership—based on Confucian culture as a conditioning factor influencing the relationship between UPB and ethical voice—fits very well into the Chinese cultural context of this study, and also allows us to gain further insight into the important role of leadership in facilitating this self-remediation process.

Finally, a new theoretical explanation is provided for the occurrence of ethical voice. Existing studies tend to explain employees’ ethical voice as social learning behaviors (e.g., Huang and Paterson, 2017; Lee et al., 2017; Kim and Vandenberghe, 2020) and social identification behaviors (e.g., Zheng et al., 2022) from the perspective of leadership moral influence. In contrast to these explanations of the mechanisms of ethical voice generation in terms of external (leadership) factors, this study finds that ethical voice is also a kind of moral compensatory behavior from the perspective of individuals’ intrinsic moral motivation. This new explanatory mechanism for the occurrence of ethical voice enhances the knowledge of ethical voice and contributes new theoretical content to its study.

### 5.2. Implications for practice

Despite their pro-organizational motives, UPB is harmful to both the organization and society in terms of their practical consequences. However, our research finds that employees who have engaged in UPB can make ethical voice for ethical remediation. Therefore, managers should pay close attention to the occurrence of UPBs in their organization; when they detect such behaviors, they should promptly help employees realize the moral implications of their actions, and encourage them to correct their mistakes and engage in compensatory ethical behaviors, such as providing ethical voice. Besides engaging in UPB, employees may also exhibit other undesirable behaviors. Managers should care for these employees and offer them help and support afterwards, so as to motivate them to make constructive behavioral changes.

Second, this study shows that UPB has a positive impact on ethical voice through moral ownership. Thus, managers can take steps to foster and enhance employees’ moral responsibility from a moral ownership perspective to promote the moral compensatory effects of UPB, such as by implementing regular formal ethics training and learning systems. Additionally, managers should create an ethical work environment that guides employees’ ethical values and inspires them to be ethically responsible.

Third, the present study also confirms that benevolent leadership plays an important role in facilitating the process of self-remediation of UPB. For this reason, supervisors should be encouraged and guided to adopt a benevolent leadership style to appropriately deal with ethically erring employees. Leaders need to help employees who engage in UPB confront their mistakes, considering that some of them may not be aware of their ethical mistakes due to the pro-organizational motivation of the unethical behavior. Furthermore, leaders should encourage morally erring employees to correct their mistakes by being tolerant rather than blaming, and by giving them the necessary guidance and resources to support them in the correction process.

Finally, ethical voice is a high-risk and high-cost ethical behavior. By providing protection and incentives for ethical voice, companies can enhance the value of ethical voice in the minds of employees, which not only can improve the moral standards within the enterprise, strengthen the employees’ moral consciousness and conduct, but also can promote the enterprise to undertake social moral responsibility more effectively, actively respond to social expectations and concerns, and achieve higher moral compensation effect of UPB.

### 5.3. Limitations and future research

This study is a prospective preliminary exploratory work aimed at discovering hitherto unexplored consequences of UPB, and has certain shortcomings. First, although this study used a three-time point approach to control for the problem of common method bias for variable measurements, and the results of the statistical analysis indicated that this problem was not serious in this study, it is still necessary to collect research data using the multi-source, multi-time point approach recommended by Podsakoff et al. (2012) in order to enhance research rigor. Second, the external validity of this study’s findings is limited by the research sample in this study, and therefore future studies are needed to expand the scope of sample collection and increase the sample size to further test the generalizability of the study’s findings.

The third is to explore alternative moral compensation mechanisms for UPB. The moral compensation options for employees are diverse, and include helping, volunteer behavior, donating, and green behavior. Therefore, future research could further examine the moral compensatory mechanisms between UPB and these positive behaviors.

Finally, this study confirms that benevolent leadership is one of the boundary conditions for the occurrence of the moral compensatory effects of UPB. However, there are still other individual and organizational factors that may influence the process of ethical remediation of UPB. One possible individual factor is the moral quality of employees. Employees with high moral standards are likely to develop higher moral ownership in the face of their wrongdoing than employees with low moral standards. Another example is that a positive moral climate in the organization may motivate employees to engage in ethical voice behavior. Therefore, future research should seek to explore the individual and organizational conditions under which employees who engage in UPB make moral reparations through ethical voice.

## 6. Conclusion

This study empirically investigates the moral compensatory effect of UPB on ethical voice. The research results show that UPB has a significant positive effect on ethical voice, that moral ownership mediates the relationship between UPB and ethical voice, and that benevolent leadership moderates both the direct and indirect effects of UPB on ethical voice through moral ownership. These research findings reveal how and when employees implement moral remediation by making ethical voice after engaging in UPB. Therefore, our research offers new insights into the consequences of UPB and the antecedents of ethical voice, and will also draw high attention to the moral remediation of morally deviant employees in this field.

### 6.1. Scale of UPB

①To help my organization, I misrepresented the truth to make my organization look good.
②To help my organization, I exaggerated the truth about my company’s products or services to customers and clients.
③To help my organization, I withheld negative information about my company or its products from customers and clients.
④To help my organization, I gave a good recommendation on the behalf of an incompetent employee in the hope that the person will become another organization’s problem instead of my own.
⑤To help my organization, I withheld issuing a refund to a customer or client accidentally overcharged.
⑥To help my organization, I concealed information from the public that could be damaging to my organization.

### 6.2. Scale of benevolent leadership

①My supervisor is often caring and attentive to me.
②My supervisor is concerned about my personal life situation.
③My supervisor not only takes care of me but also of my family members.
④My supervisor is careful and thoughtful to subordinates who have served under him/her for a long time.
⑤My supervisor helps me in an emergency.

### 6.3. Scale of moral ownership

①I will not accept unethical behavior from anyone in my organization.
②I will assume responsibility to take action when I see an unethical act.
③I will take charge to address ethical issues when I know someone has done something wrong.

### 6.4. Scale of ethical voice

①I am prepared to talk to coworkers who fail to behave ethically.
②I would tell a coworker who is doing something unethical to stop.
③I encourage my coworkers to act with integrity.
④I speak up in our company to stop others from behaving with a lack of integrity.

## Data availability statement

The raw data supporting the conclusions of this article will be made available by the authors, without undue reservation.

## Ethics statement

The studies involving human participants were reviewed and approved by Hanshan Normal University Ethics Committee. The patients/participants provided their written informed consent to participate in this study.

## Author contributions

FX, PL, LW, and JB contributed to the study conception and design, data collection and analysis, and drafting and revising the manuscript. All authors contributed to the article and approved the submitted version.

## Funding

This work was supported by the Social Science Foundation of Heilongjiang Province (21SHB105) and the Hanshan Normal University (XS202002, QD202223, and QD202240).

## Conflict of interest

The authors declare that the research was conducted in the absence of any commercial or financial relationships that could be construed as a potential conflict of interest.

## Publisher’s note

All claims expressed in this article are solely those of the authors and do not necessarily represent those of their affiliated organizations, or those of the publisher, the editors and the reviewers. Any product that may be evaluated in this article, or claim that may be made by its manufacturer, is not guaranteed or endorsed by the publisher.

## References

[ref1] Babalola M. T. Mawritz M. B. Greenbaum R. L. Ren S. Garba O. A. (2021). Whatever it takes: how and when supervisor bottom-line mentality motivates employee contributions in the workplace. J. Manag. 47, 1134–1154. doi: 10.1177/0149206320902521

[ref2] Baker B. Derfler-Rozin R. Pitesa M. Johnson M. (2019). Stock market responses to unethical behavior in organizations: an organizational context model. Organ. Sci. 30, 319–336. doi: 10.1287/orsc.2018.1244

[ref3] Baron R. M. Kenny D. A. (1986). The moderator-mediator variable distinction in social psychological research: conceptual, strategic, and statistical considerations. J. Pers. Soc. Psychol. 51, 1173–1182. doi: 10.1037/0022-3514.51.6.1173, PMID: 3806354

[ref4] Bateson M. Nettle D. Roberts G. (2006). Cues of being watched enhance cooperation in a real-world setting. Biol. Lett. 2, 412–414. doi: 10.1098/rsbl.2006.0509, PMID: 17148417 PMC1686213

[ref5] Baur C. Soucek R. Kuehnen U. Baumeister R. F. (2020). Unable to resist the temptation to tell the truth or to lie for the organization? Identification makes the difference. J. Bus. Ethics 167, 643–662. doi: 10.1007/s10551-019-04162-3

[ref6] Blau P. M. (1964). Exchange and power in social life. New York: John Wiley and Sons.

[ref7] Bolin J. H. (2014). Introduction to mediation, moderation, and conditional process analysis: a regression-based approach. J. Educ. Meas. 51, 335–337. doi: 10.1111/jedm.12050

[ref8] Bonner J. M. Greenbaum R. L. Quade M. J. (2017). Employee unethical behavior to shame as an indicator of self-image threat and exemplification as a form of self-image protection: the exacerbating role of supervisor bottom-line mentality. J. Appl. Psychol. 102, 1203–1221. doi: 10.1037/apl0000222, PMID: 28383944

[ref9] Brislin R. W. (1986). A culture general assimilator: preparation for various types of sojourns. Int. J. Intercult. Relat. 10, 215–234. doi: 10.1016/0147-1767(86)90007-6

[ref10] Chan S. C. H. Mak W.-M. (2012). Benevolent leadership and follower performance: the mediating role of leader-member exchange (lmx). Asia Pacific J. Manag. 29, 285–301. doi: 10.1007/s10490-011-9275-3

[ref11] Chen X.-P. Eberly M. B. Chiang T.-J. Farh J.-L. Cheng B.-S. (2014). Affective trust in Chinese leaders: linking paternalistic leadership to employee performance. J. Manag. 40, 796–819. doi: 10.1177/0149206311410604

[ref12] Chen H. Kwan H. K. Xin J. (2022). Is behaving unethically for organizations a mixed blessing? A dual-pathway model for the work-to-family spillover effects of unethical pro-organizational behavior. Asia Pacific J. Manag. 39, 1535–1560. doi: 10.1007/s10490-021-09776-8

[ref13] Cheng B.-S. Chou L. F. Farh J.-L. (2000). A triad model of paternalistic leadership: the constructs and measurement. Indig. Psychol. Res. Chin. Soc. 14, 3–64. doi: 10.6254/IPRCS

[ref14] Cheng B.-S. Chou L.-F. Wu T.-Y. Huang M.-P. Farh J.-L. (2004). Paternalistic leadership and subordinate responses: establishing a leadership model in Chinese organizations. Asian J. Soc. Psychol. 7, 89–117. doi: 10.1111/j.1467-839X.2004.00137.x

[ref15] Cheng K. Guo L. Lin Y. Hu P. Hou C. He J. (2022). Standing in customers' shoes: how responsible leadership inhibits unethical pro-organizational behavior. Front. Psychol. 13:1019734. doi: 10.3389/fpsyg.2022.1019734, PMID: 36524194 PMC9744943

[ref16] Chua R. Y. J. Ingram P. Morris M. W. (2008). From the head and the heart: locating cognition- and affect-based trust in managers' professional networks. Acad. Manage. J. 51, 436–452. doi: 10.5465/amj.2008.32625956

[ref17] Cornelissen G. Bashshur M. R. Rode J. Le Menestrel M. (2013). Rules or consequences? The role of ethical mind-sets in moral dynamics. Psychol. Sci. 24, 482–488. doi: 10.1177/0956797612457376 23447556

[ref18] Effelsberg D. Solga M. Gurt J. (2014). Transformational leadership and follower's unethical behavior for the benefit of the company: a two-study investigation. J. Bus. Ethics 120, 81–93. doi: 10.1007/s10551-013-1644-z

[ref19] Epitropaki O. Kark R. Mainemelis C. Lord R. G. (2017). Leadership and followership identity processes: a multilevel review. Leadersh. Q. 28, 104–129. doi: 10.1016/j.leaqua.2016.10.003

[ref20] Farasat M. Azam A. (2022). Supervisor bottom-line mentality and subordinates' unethical pro-organizational behavior. Pers. Rev. 51, 353–376. doi: 10.1108/pr-03-2020-0129

[ref21] Farh J.-L. Cheng B.-S. (2000). “A cultural analysis of paternalistic leadership in Chinese organizations” in Management and organizations in the Chinese context. eds. Li J. T. Tsui A. S. Weldon E. (London: Palgrave Macmillan UK), 84–127.

[ref22] Farh J. L. Liang J. Chou L. F. Cheng B. S. (2008). “Paternalistic leadership in Chinese organizations: research progress and future research directions” in Management in China: Philosophies, theories, practices. eds. Chen C. C. Lee Y. T. (London: Cambridge University Press), 171–205.

[ref23] Fehr R. Welsh D. Yam K. C. Baer M. Wei W. Vaulont M. (2019). The role of moral decoupling in the causes and consequences of unethical pro-organizational behavior. Organ. Behav. Hum. Decis. Process. 153, 27–40. doi: 10.1016/j.obhdp.2019.05.007

[ref24] Fu X. Li Y. Si Y. (2012). The impact of paternalistic leadership on innovation: an integrated model. Nankai Bus. Rev. 15, 121–127.

[ref25] Gino F. (2015). Understanding ordinary unethical behavior: why people who value morality act immorally. Curr. Opin. Behav. Sci. 3, 107–111. doi: 10.1016/j.cobeha.2015.03.001

[ref26] Gino F. Schweitzer M. E. Mead N. L. Ariely D. (2011). Unable to resist temptation: how self-control depletion promotes unethical behavior. Organ. Behav. Hum. Decis. Process. 115, 191–203. doi: 10.1016/j.obhdp.2011.03.001

[ref27] Gollwitzer P. M. Wicklund R. A. Hilton J. L. (1982). Admission of failure and symbolic self-completion: extending Lewinian theory. J. Pers. Soc. Psychol. 43, 358–371. doi: 10.1037/0022-3514.43.2.358

[ref28] Graen G. B. Uhl-Bien M. (1995). Relationship-based approach to leadership: development of leader-member exchange (lmx) theory of leadership over 25 years: applying a multi-level multi-domain perspective. Leadersh. Q. 6, 219–247. doi: 10.1016/1048-9843(95)90036-5

[ref29] Graham K. A. Resick C. J. Margolis J. A. Shao P. Hargis M. B. Kiker J. D. (2020). Egoistic norms, organizational identification, and the perceived ethicality of unethical pro-organizational behavior: a moral maturation perspective. Hum. Relat. 73, 1249–1277. doi: 10.1177/0018726719862851

[ref30] Greene M. Low K. (2014). Public integrity, private hypocrisy, and the moral licensing effect. Soc. Behav. Pers. 42, 391–400. doi: 10.2224/sbp.2014.42.3.391

[ref31] Gumusluoglu L. Karakitapoglu-Aygun Z. Scandura T. A. (2017). A multilevel examination of benevolent leadership and innovative behavior in R&D contexts: a social identity approach. J. Leadersh. Organ. Stud. 24, 479–493. doi: 10.1177/1548051817705810

[ref32] Hannah S. T. Avolio B. J. (2010). Moral potency: building the capacity for character-based leadership. Consult. Psychol. J.: Pract. Res. 62, 291–310. doi: 10.1037/a0022283

[ref33] Hannah S. T. Avolio B. J. May D. R. (2011). Moral maturation and moral conation: a capacity approach to explaining moral thought and action. Acad. Manage. Rev. 36, 663–685. doi: 10.5465/amr.2010.0128

[ref34] Huang Y. Liu X. Kim J. Na S. (2022). Effects of idiosyncratic deals, psychological contract, job satisfaction and environmental turbulence on unethical pro-organizational behavior. Sustainability 14:15995. doi: 10.3390/su142315995

[ref35] Huang L. Paterson T. A. (2017). Group ethical voice: influence of ethical leadership and impact on ethical performance. J. Manag. 43, 1157–1184. doi: 10.1177/0149206314546195

[ref36] Jino M. J. Dyaram L. (2019). The mediating role of moral ownership in the relationship between organizational support and employees’ ethical behavior: a study of higher education faculty members. Ethics Behav. 29, 305–319. doi: 10.1080/10508422.2017.1409628

[ref37] Johnson H. H. Umphress E. E. (2019). To help my supervisor: identification, moral identity, and unethical pro-supervisor behavior. J. Bus. Ethics 159, 519–534. doi: 10.1007/s10551-018-3836-z

[ref38] Jones T. M. Ryan L. V. (1997). The link between ethical judgment and action in organizations: a moral approbation approach. Organ. Sci. 8, 663–680. doi: 10.1287/orsc.8.6.663

[ref39] Jordan J. Mullen E. Murnighan J. K. (2011). Striving for the moral self: the effects of recalling past moral actions on future moral behavior. Pers. Soc. Psychol. Bull. 37, 701–713. doi: 10.1177/0146167211400208, PMID: 21402752

[ref40] Karakas F. Sarigollu E. (2012). Benevolent leadership: conceptualization and construct development. J. Bus. Ethics 108, 537–553. doi: 10.1007/s10551-011-1109-1

[ref41] Kelebek E. E. Alniacik E. (2022). Effects of leader-member exchange, organizational identification and leadership communication on unethical pro-organizational behavior: a study on bank employees in Turkey. Sustainability 14:1055. doi: 10.3390/su14031055

[ref42] Kim D. Vandenberghe C. (2020). Ethical leadership and team ethical voice and citizenship behavior in the military: the roles of team moral efficacy and ethical climate. Group Org. Manag. 45, 514–555. doi: 10.1177/1059601120920050

[ref43] Klotz A. C. Bolino M. C. (2013). Citizenship and counterproductive work behavior: a moral licensing view. Acad. Manage. Rev. 38, 292–306. doi: 10.5465/amr.2011.0109

[ref44] Lee D. Choi Y. Youn S. Chun J. U. (2017). Ethical leadership and employee moral voice: the mediating role of moral efficacy and the moderating role of leader-follower value congruence. J. Bus. Ethics 141, 47–57. doi: 10.1007/s10551-015-2689-y

[ref45] Lee A. Schwarz G. Newman A. Legood A. (2019). Investigating when and why psychological entitlement predicts unethical pro-organizational behavior. J. Bus. Ethics 154, 109–126. doi: 10.1007/s10551-017-3456-z

[ref46] Li G. Rubenstein A. L. Lin W. Wang M. Chen X. (2018). The curvilinear effect of benevolent leadership on team performance: the mediating role of team action processes and the moderating role of team commitment. Pers. Psychol. 71, 369–397. doi: 10.1111/peps.12264

[ref47] Lian H. Huai M. Farh J.-L. Huang J.-C. Lee C. Chao M. M. (2022). Leader unethical pro-organizational behavior and employee unethical conduct: social learning of moral disengagement as a behavioral principle. J. Manag. 48, 350–379. doi: 10.1177/0149206320959699

[ref48] Lin W. Ma J. Zhang Q. Li J. C. Jiang F. (2018). How is benevolent leadership linked to employee creativity? The mediating role of leader-member exchange and the moderating role of power distance orientation. J. Bus. Ethics 152, 1099–1115. doi: 10.1007/s10551-016-3314-4

[ref49] Liu X. L. Lu J. G. Zhang H. Cai Y. (2021). Helping the organization but hurting yourself: how employees’ unethical pro-organizational behavior predicts work-to-life conflict. Organ. Behav. Hum. Decis. Process. 167, 88–100. doi: 10.1016/j.obhdp.2021.05.002

[ref50] Loi T. I. Kuhn K. M. Sahaym A. Butterfield K. D. Tripp T. M. (2020). From helping hands to harmful acts: when and how employee volunteering promotes workplace deviance. J. Appl. Psychol. 105, 944–958. doi: 10.1037/apl0000477 31904249

[ref51] Martin R. Guillaume Y. Thomas G. Lee A. Epitropaki O. (2016). Leader-member exchange (lmx) and performance: a meta-analytic review. Pers. Psychol. 69, 67–121. doi: 10.1111/peps.12100

[ref52] Mo S. Lupoli M. J. Newman A. Umphress E. E. (2022). Good intentions, bad behavior: a review and synthesis of the literature on unethical prosocial behavior (upb) at work. J. Organ. Behav., in press 44, 335–354. doi: 10.1002/job.2617

[ref53] Nguyen C. M. Zhang L. Morand D. (2021). Unethical pro-organizational behavior: a moderated mediational model of its transmission from managers to employees. J. Leadersh. Organ. Stud. 28, 379–393. doi: 10.1177/15480518211005464

[ref54] Nisan M. Horenczyk G. (1990). Moral balance: the effect of prior behaviour on decision in moral conflict. Br. J. Soc. Psychol. 29, 29–42. doi: 10.1111/j.2044-8309.1990.tb00884.x 2322781

[ref55] Nunnally J. C. (1978). Psychometric theory (2nd Edn.). New York: McGraw-Hill.

[ref56] Ogunfowora B. Maerz A. Varty C. T. (2021). How do leaders foster morally courageous behavior in employees? Leader role modeling, moral ownership, and felt obligation. J. Organ. Behav. 42, 483–503. doi: 10.1002/job.2508

[ref57] Paterson T. A. Huang L. (2019). Am I expected to be ethical? A role-definition perspective of ethical leadership and unethical behavior. J. Manag. 45, 2837–2860. doi: 10.1177/0149206318771166

[ref58] Podsakoff P. M. MacKenzie S. B. Lee J.-Y. Podsakoff N. P. (2003). Common method biases in behavioral research: a critical review of the literature and recommended remedies. J. Appl. Psychol. 88, 879–903. doi: 10.1037/0021-9010.88.5.879, PMID: 14516251

[ref59] Podsakoff P. M. MacKenzie S. B. Podsakoff N. P. (2012). Sources of method bias in social science research and recommendations on how to control it. Annu. Rev. Psychol. 63, 539–569. doi: 10.1146/annurev-psych-120710-100452 21838546

[ref60] Pyszczynski T. Greenberg J. Solomon S. Arndt J. Schimel J. (2004). Why do people need self-esteem? A theoretical and empirical review. Psychol. Bull. 130, 435–468. doi: 10.1037/0033-2909.130.3.435, PMID: 15122930

[ref61] Sachdeva S. Iliev R. Medin D. L. (2009). Sinning saints and saintly sinners: the paradox of moral self-regulation. Psychol. Sci. 20, 523–528. doi: 10.1111/j.1467-9280.2009.02326.x 19320857

[ref62] Sheedy E. Garcia P. Jepsen D. (2021). The role of risk climate and ethical self-interest climate in predicting unethical pro-organisational behaviour. J. Bus. Ethics 173, 281–300. doi: 10.1007/s10551-020-04542-0

[ref63] Su X. Jiang X. Xie G. Huang M. Xu A. (2022). How does self-sacrificial leadership foster knowledge sharing behavior in employees? Moral ownership, felt obligation and supervisor-subordinate guanxi. Front. Psychol. 13:910707. doi: 10.3389/fpsyg.2022.910707, PMID: 35899007 PMC9309226

[ref64] Tang C. Naumann S. E. (2015). Paternalistic leadership, subordinate perceived leader-member exchange and organizational citizenship behavior. J. Manag. Organ. 21, 291–306. doi: 10.1017/jmo.2014.84

[ref65] Tang T. L.-P. Sutarso T. (2013). Falling or not falling into temptation? Multiple faces of temptation, monetary intelligence, and unethical intentions across gender. J. Bus. Ethics 116, 529–552. doi: 10.1007/s10551-012-1475-3

[ref66] Tang P. M. Yam K. C. Koopman J. (2020). Feeling proud but guilty? Unpacking the paradoxical nature of unethical pro-organizational behavior. Organ. Behav. Hum. Decis. Process. 160, 68–86. doi: 10.1016/j.obhdp.2020.03.004

[ref67] Umphress E. E. Bingham J. B. (2011). When employees do bad things for good reasons: examining unethical pro-organizational behaviors. Organ. Sci. 22, 621–640. doi: 10.1287/orsc.1100.0559

[ref68] Umphress E. E. Bingham J. B. Mitchell M. S. (2010). Unethical behavior in the name of the company: the moderating effect of organizational identification and positive reciprocity beliefs on unethical pro-organizational behavior. J. Appl. Psychol. 95, 769–780. doi: 10.1037/a0019214, PMID: 20604596

[ref69] Valle M. Kacmar K. M. Zivnuska S. (2019). Understanding the effects of political environments on unethical behavior in organizations. J. Bus. Ethics 156, 173–188. doi: 10.1007/s10551-017-3576-5

[ref70] Wang A.-C. Cheng B.-S. (2010). When does benevolent leadership lead to creativity? The moderating role of creative role identity and job autonomy. J. Organ. Behav. 31, 106–121. doi: 10.1002/job.634

[ref71] Wang Y. Xiao S. Ren R. (2022). A moral cleansing process: how and when does unethical pro-organizational behavior increase prohibitive and promotive voice. J. Bus. Ethics 176, 175–193. doi: 10.1007/s10551-020-04697-w, PMID: 33437107 PMC7791148

[ref72] Yam K. C. Klotz A. C. He W. Reynolds S. J. (2017). From good soldiers to psychologically entitled: examining when and why citizenship behavior leads to deviance. Acad. Manage. J. 60, 373–396. doi: 10.5465/amj.2014.0234

[ref73] Zhang Y. He B. Sun X. (2018). The contagion of unethical pro-organizational behavior: from leaders to followers. Front. Psychol. 9:1102. doi: 10.3389/fpsyg.2018.01102, PMID: 30018583 PMC6038011

[ref74] Zhang H. Liu X. L. Cai Y. Sun X. (2022). Paved with good intentions: self-regulation breakdown after altruistic ethical transgression. J. Bus. Ethics. doi: 10.1007/s10551-022-05185-z

[ref75] Zheng Y. Epitropaki O. Graham L. Caveney N. (2022). Ethical leadership and ethical voice: the mediating mechanisms of value internalization and integrity identity. J. Manag. 48, 973–1002. doi: 10.1177/01492063211002611

[ref76] Zheng Y. Graham L. Farh J.-L. Huang X. (2021). The impact of authoritarian leadership on ethical voice: a moderated mediation model of felt uncertainty and leader benevolence. J. Bus. Ethics 170, 133–146. doi: 10.1007/s10551-019-04261-1

